# Antibiofilm and Anti-Infection of a Marine Bacterial Exopolysaccharide Against *Pseudomonas aeruginosa*

**DOI:** 10.3389/fmicb.2016.00102

**Published:** 2016-02-08

**Authors:** Shimei Wu, Ge Liu, Weihua Jin, Pengyuan Xiu, Chaomin Sun

**Affiliations:** ^1^Key Laboratory of Biobased Materials, Qingdao Institute of Bioenergy and Bioprocess Technology, Chinese Academy of SciencesQingdao, China; ^2^Key Laboratory of Experimental Marine Biology, Institute of Oceanology, Chinese Academy of SciencesQingdao, China; ^3^Laboratory for Marine Biology and Biotechnology, Qingdao National Laboratory for Marine Science and TechnologyQingdao, China; ^4^University of Chinese Academy of SciencesBeijing, China

**Keywords:** antibiofilm, anti-infection, marine, exopolysaccharide, *Pseudomonas*

## Abstract

*Pseudomonas aeruginosa* is a well-known pathogenic bacterium that forms biofilms and produces virulence factors, thus leading to major problems in many fields, such as clinical infection, food contamination, and marine biofouling. In this study, we report the purification and characterization of an exopolysaccharide EPS273 from the culture supernatant of marine bacterium *P. stutzeri* 273. The exopolysaccharide EPS273 not only effectively inhibits biofilm formation but also disperses preformed biofilm of *P. aeruginosa* PAO1. High performance liquid chromatography traces of the hydrolyzed polysaccharides shows that EPS273 primarily consists of glucosamine, rhamnose, glucose and mannose. Further investigation demonstrates that EPS273 reduces the production of the virulence factors pyocyanin, exoprotease, and rhamnolipid, and the virulence of *P. aeruginosa* PAO1 to human lung cells A549 and zebrafish embryos is also obviously attenuated by EPS273. In addition, EPS273 also greatly reduces the production of hydrogen peroxide (H_2_O_2_) and extracellular DNA (eDNA), which are important factors for biofilm formation. Furthermore, EPS273 exhibits strong antioxidant potential by quenching hydroxyl and superoxide anion radicals. Notably, the antibiofouling activity of EPS273 is observed in the marine environment up to 2 weeks according to the amounts of bacteria and diatoms in the glass slides submerged in the ocean. Taken together, the properties of EPS273 indicate that it has a promising prospect in combating bacterial biofilm-associated infection, food-processing contamination and marine biofouling.

## Introduction

*Pseudomonas aeruginosa* is a Gram-negative, rod-shaped bacterium that belongs to the family *Pseudomonadaceae*, which is widespread in nature, inhabiting soil, water, plant, animals, and human beings (Franzetti and Scarpellini, 2007). It is an opportunistic pathogen and rarely cause disease in healthy persons, but is a notorious nosocomial pathogen, posing a high risk to immunosuppressed individuals and other highly vulnerable patients (Balasubramanian et al., 2013). *P. aeruginosa* can cause pneumonia, catheter-associated and urinary tract infections, and sepsis in wounded patients, sometimes resulting in serious chronic infections and health complications. In particular, it is fatal to patients with cystic fibrosis by forming mucoid in lung tissue (Bjarnsholt et al., 2009). In addition, *P. aeruginosa* has also been implicated as the most common bacterial pathogen in marine aquaculture (Thomas et al., 2014), and is considered as the causes of spoilage in aquatic product (Gram and Huss, 1996). Furthermore, *P. aeruginosa* is also a major food spoilage microorganism in food processing industry, such as in brewing, dairy processing, fresh produce, poultry processing, and red meat processing (Srey et al., 2013). The worst of all, *P. aeruginosa* can colonize on various surfaces by forming a biofilm (Flemming and Wingender, 2010), and the cells in biofilm are more resistant to antibiotics and biocides than planktonic cells, thus causing difficulties in eradicating them completely (Hoyle and Costerton, 1991).

Biofilm is a bacteria community which adhere to biotic and abiotic surface and embedded in a polymeric matrix composed mainly of polysaccharides, proteins, nucleic acids (Flemming and Wingender, 2010). Biofilm is the predominant mode of growth for bacteria in natural, clinical, industrial and food-processing environments. It is now recognized that many outbreaks of pathogens have been found to be associated with biofilms (Lapidot et al., 2006; Aarnisalo et al., 2007), and bacterial biofilms are accounting for over 80% of microbial infections in the body (Moreau-Marquis et al., 2008). Within biofilms bacteria are generally well protected against the influence of disinfectants, antibiotics and the host immune system. Compared with the planktonic ones, bacteria within biofilm are upwards of 1000-times more resistant to conventional antibiotic treatment and host immune responses, leading to biofilms extremely difficult to eradicate (Burmolle et al., 2010). Therefore, searching for novel compounds or strategies to inhibit biofilm formation or disperse preformed biofilm is needed.

Biofilm is also the root cause of biofouling in most cases (Dafforn et al., 2011). Biofouling in aquaculture is a specific problem where both the target culture species and/or infrastructure are exposed to a diverse array of fouling organisms, with significant production impacts (Fitridge et al., 2012). To combat biofouling, one strategy is to control biofilm development during the first step of fouling adhesion by antifouling paints, but the traditional antifouling paints are toxic and have the potential to cause environmental problem (Dafforn et al., 2011; Wu et al., 2015). Therefore, it is essential to look for environmental friendly antibiofilm agents to combat biofouling.

It is well known that the biodiversity of the marine environment and the associated chemical diversity constitute a practically unlimited resource of new bioactive substances (Carte, 1993), and the bioactive compounds from marine microorganisms have been exploited for decades (Jiang et al., 2011). However, their application in treating detrimental biofilms is still a relatively less-explored area (Miao and Qian, 2005). In this study, we show that an exopolysaccharide EPS273 purified from culture supernatant of the marine bacterium *P. stutzeri* 273 not only inhibits biofilm formation but also disrupts the established biofilms of *P. aeruginosa* PAO1. EPS273 also increases the survival rates of human lung cells and zebrafish embryos in the presence of *P. aeruginosa* PAO1. Furthermore, the strong antioxidant and antibiofouling activities of EPS273 are also reported.

## Materials and Methods

### Bacterial Strains Isolation, Identification and Culture Conditions

The marine bacteria strains used in this study were isolated from the sediments of East China Sea and cultured in marine broth 2216E (5 g/L tryptone, 1 g/L yeast extract, one liter filtered seawater, pH adjusted to 7.4–7.6) or Luria Bertani (LB) medium (10 g/L peptone, 5 g/L yeast extract, 10 g/L NaCl, pH adjusted to 7.0), and incubated at 28°C. Genomic DNA was extracted from the isolate, and PCR (polymerase chain reaction) was performed to amplify the 16S rRNA gene sequence with universal primers 27F (AGAGTTTGATCCTGGCTCAG) and 1541R (AAGGAGGTGATCCACCC). The 16S rRNA gene sequence was analyzed by using the BLAST programs^1^ to determine the phylogenetic position of the bacterium strain 273. *P. aeruginosa* PAO1 was cultured in LB medium and incubated at 37°C.

### Purification of Antibiofilm Component EPS273 from Marine Bacterium *Pseudomonas Stuzeri* 273

The marine bacterium *P. stutzeri* 273 was cultured in glass flasks containing LB medium and incubated at 28°C under vigorous agitation for 48 h. Cell-free culture supernatant of the strain *P. stutzeri* 273 was collected by centrifugation at 12,000 rpm for 15 min, and precipitated overnight at 80% saturation with (NH_4_)_2_SO_4_ at 4°C. The precipitate was collected by centrifugation and dissolved in 50 mM NaCl in 20 mM Tris-HCl (pH 9.0), and dialyzed against the same buffer overnight. The dialyzed fraction was loaded onto a 5 mL HiTrap^TM^ Q HP column (GE Healthcare) pre-equilibrated with 50 mM NaCl in 20 mM Tris-HCl (pH 9.0), then eluted with a NaCl gradient (50-500 mM) in the same buffer at 5 mL/min. Active fractions were collected, concentrated by ultra-filtration (100-kDa MW cut-off membrane, Millipore), and subjected to gel filtration on a Hiload^TM^ 16/600 Superdex^TM^ 200 column (GE Healthcare) pre-equilibrated with 150 mM NaCl in 20 mM Tris-HCl (pH 9.0). The column was eluted with the same buffer at a flow rate of 1 mL/min, and the active fractions were pooled for further analysis. All purification was performed at 4°C. Total sugar content was determined by the phenol-sulfuric acid method using glucose as the standard (Jiang et al., 2011), and active fractions were determined as described in the following section of inhibition assay of biofilm formation.

### The Inhibition Assay of Biofilm Formation with Supernatant of *P. stutzeri* 273

The inhibition assay of biofilm formation was performed according to the method as described previously (Sambanthamoorthy et al., 2014; Wu et al., 2015). Briefly, the overnight culture of *P. aeruginosa* PAO1 was diluted with LB medium to OD_600_ of 0.1, and incubated statically in 96-well polystyrene plate with or without 10% (volume/volume) supernatant of *P. stutzeri* 273 at 37°C for 24 h. For the quantification of biofilm, planktonic bacteria were discarded, and the wells were rinsed gently with sterile distilled water, air-dried for 10 min and stained with 1% crystal violet for 10 min. The stained biofilm was washed again with sterile distilled water followed by the addition of 200 μl of ethanol (95%, volume/volume) to dissolve the crystal violet, and the absorbance was determined at 595 nm.

We also checked the inhibition activity on rectangular glass slides, which were placed in 12-well polystyrene plate containing LB broth with OD_600_ about 0.1 *P. aeruginosa* PAO1. The wells containing the glass slides were supplemented with or without 10% (volume/volume) supernatant of *P. stutzeri* 273 and incubated at 37°C. The glass slides were taken out and gently washed with sterile water after incubated for 24 h. Biofilms on the glass slides were stained with crystal violet as described above and recorded with photograph.

### The Dispersion Assay of Preformed Biofilm with Supernatant of *P. stutzeri* 273

For the biofilm dispersion assay, the overnight culture of *P. aeruginosa* PAO1 was diluted with LB medium to OD_600_ of 0.1, and incubated statically in 96-well polystyrene plate at 37°C to allow the biofilm formation. Following the biofilm formation after incubation for 24 h, the biofilm was treated with or without 10% (volume/volume) supernatant of *P. stutzeri* 273 and incubated overnight. The biofilm quantification was performed using the crystal violet staining technique according to the method as described above.

We also checked the dispersion activity on rectangular glass slides, which were placed in 12-well polystyrene plate containing LB broth with OD_600_ about 0.1 *P. aeruginosa* PAO1. After the biofilms formed on glass pieces at 37°C for 24 h, the wells containing the glass slides were supplemented with 10% (volume/volume) supernatant of *P. stutzeri* 273 and incubated overnight. The glass slides were taken out and gently washed with sterile water. Biofilms on the glass slides were stained with crystal violet and recorded with photograph.

### Physical and Chemical Analysis of Antibiofilm Component of EPS273

For enzymatic treatment, the purified EPS273 aliquots with a final concentration of 0.1 μg/mL were incubated at 37°C for 1 h with 100 μg/mL DNase I, RNase A or 1 mg/mL proteinase K (Sigma–Aldrich). For sodium metaperiodate treatment, EPS273 aliquot with a final concentration of 0.1 μg/mL was incubated at with 10 mM sodium metaperiodate 37°C for 12 h. As controls, EPS273 aliquots were incubated at 37°C for corresponding time in the absence of enzymes or sodium metaperiodate. For each of the above tests, the antibiofilm activities of EPS273 aliquots with or without treatment were compared using 96-well polystyrene plate assay against *P. aeruginosa* PAO1.

For infrared (IR) spectroscopy, as previously described (Sun et al., 2009), EPS273 was mixed with KBr powder, ground and then pressed into a 1 mm pellets for fourier transform infrared (FT-IR) measurement in the frequency range of 4000–500 cm^-1^, FT-IR spectra of EPS273 was measured on a Nicolet Nexus 470 spectrometer. The molecular weight was assessed by High Performance Gel Permeation Chromatography (HPGPC; Sun et al., 2009). The monosaccharide components were determined by High-performance liquid chromatography (HPLC; Sun et al., 2009). The standard saccharides used here were glucose, galactose, mannose, rhamnose, xylose, glucuronic acid, galacturonic acid, and glucosamine.

### Fluorescence Microscopic Observation of Biofilm Inhibition and Dispersion by EPS273

For the inhibition assay of biofilm formation, the wells of polystyrene plate containing rectangular glass slides and LB broth with OD600 about 0.1 *P. aeruginosa* PAO1 were supplemented with or without 0.1 μg/mL, 0.5 μg/mL of purified EPS273 and incubated at 37°C for 24 h. Biofilms on the glass slides were stained with 0.01% acridine orange solution for 5 min and observed under fluorescence microscope (Imaginer A2, Zeiss, Germany).

For the dispersion assay of the preformed biofilm, after the biofilm formed on the glass slides in the 12-well polystyrene plate, the wells containing the glass slides were supplemented with or without 0.1 μg/mL, 0.5 μg/mL of purified EPS273 and incubated at 37°C overnight. Biofilms on the glass slides were stained with 0.01% acridine orange solution for 5 min and observed under fluorescence microscope (Imaginer A2, Zeiss, Germany).

### Antibiofilm and Antimicrobial Assays with EPS273

The antibiofilm and antimicrobial activities of purified EPS273 on *P. aeruginosa* PAO1 were checked in 96-well polystyrene microplate according to the method described previously with minor modification (Sambanthamoorthy et al., 2014; Wu et al., 2015). Briefly, the overnight culture of *P. aeruginosa* PAO1 was diluted with LB medium to OD600 of 0.1, and incubated statically in 96-well polystyrene plate with or without 10% cell-free supernatant, 0.1 μg/mL or 0.5 μg/mL purified EPS273, respectively, at 37°C for 24 h. For the biofilm quantification, the biofilm was stained with crystal violet. For the planktonic cells, the contents of each well were plated onto LB agar plates with appropriate dilutions, and colony-forming unit (cfu) were enumerated the next day. The biofilm or the cfu of *P. aeruginosa* PAO1 incubated with the same amount of buffer was set as 100%.

### Effects of EPS273 on Production of Virulence Factors, H_2_O_2_ and eDNA by *P. aeruginosa* PAO1

*Pseudomonas aeruginosa* PAO1 was inoculated in glass flasks containing LB broth without or with EPS273 (final concentration at 0.1 μg/mL), and incubated at 37°C statically. At different growth times, *P. aeruginosa* PAO1 cells were separated from culture via centrifugation at 12, 000 rpm for 15 min, and the culture supernatants were passed through 0.22-μm syringe-driven filters (Millipore).

The quantity of pyocyanin was measured according to the method described previously (Musthafa et al., 2012). Briefly, crude pyocyanin was initially extracted by mixing 5 mL cell-free supernatants with 3 mL chloroform. The pyocyanin in the layer of chloroform was then re-extracted with 1 mL 0.2 N HCl to get a pink to deep-red solution. The quantity of pyocyanin was measured at 520 nm using spectrophotometer.

The proteolytic activity was determined according to the method described previously with some modification (Kessler et al., 1993). Briefly, 200 μl of cell free supernatant was mixed with 50 μl of 0.3% azocasein in 0.05 M Tris-HCl (pH7.5), and the reaction mixture was subsequently incubated at 37°C for 60 min. The reaction was stopped by the addition of 10% trichloroacetic acid. Exoprotease activity was measured at 440 nm by spectrophotometer.

For assaying rhamnolipid activity, 5 mL cell-free supernatant was acidified to pH 2.0 with concentrated HCl, and then two volumes of chloroform/ethanol (2:1, v/v) were added to extract rhamnolipid by vigorous mixing. The organic phase was evaporated to dryness, and the rhamnolipid residue was dissolved in 1 mL deionized water. The quantity of rhmnolipid was measured according to the method described previously (Kim et al., 2015). Briefly, 100 μl rhamnolipid solution was mixed with 900 μl orcinal solution (0.19% orcinal (Sigma–Aldrich) in 53% H_2_SO_4_. The mixture was boiled for 30 min, and then cooled at room temperature for 15 min. Rhamnolipid activity was measured at 421 nm using spectrophotometer.

For H_2_O_2_ generation, a colorimetric assay was applied as described previously (Das and Manefield, 2012). Briefly, the freshly prepared solution of 160 μl of sodium acetate (0.1 M) containing 0.1 μg of horseradish peroxidase and 10 μl of 1 mg/mL o-dianisidine in methanol, 40 μl of cell free supernatant was added in 96-wells microtiter plate and incubated for 10 min at room temperature protected from light. The absorbance of H_2_O_2_ in the mixture solution was determined using a microplate reader at 570 nm.

For the quantification of eDNA, 50 μl of cell free supernatants were incubated with 50 μl of Quant-it Picogreen reagent (Invitrogen). The mixture was excited at 485 nm and fluorescence intensity was measured at 520 nm. The measured amount of eDNA was determined by the standard curve according to the quant-it picogreen ds DNA kit (Invitrogen).

### Inhibition Activities of EPS273 on *P. aeruginosa* PAO1 Infection Toward Human Lung Carcinosis A549 Cells

*Pseudomonas aeruginosa* PAO1 infection assay was performed according to O’Loughlin et al. (2013) with minor modification. Human A549 cells were grown in CellStar tissue culture flasks (Nest, China). Before infection, the A549 cells were treated with trypsin-EDTA, split, counted, aliquoted into 96-well plates at 20,000 mammalian cells per well, and incubated at 37°C in the presence of 5% CO_2_. After incubated for 24 h, cells were washed three times with pre-warmed PBS before 90 μl of PBS containing propidium iodide (10 μg/mL) was added to each well, then 10 μl of OD_600_ > 1.0 *P. aeruginosa* PAO1 preincubated or non-preincubated with different concentrations of EPS273 (0.1 μg/mL, 0.5 μg/mL) was added. In addition, 10 μl of OD_600_ > 1.0 *P. aeruginosa* PAO1 non-preincubated with EPS273 was also added parallel with different concentrations of EPS273 (0.1 μg/mL, 0.5 μg/mL). Cells were further incubated at 37°C, and infections were monitored using a microplate reader after incubated for 8 h with Red FP filter. For the cytotoxic assay of EPS273 to human cells, different concentration of EPS273 (0.1, 0.5, and 1 μg/mL) was added parallel to human A549 cells, and cells were incubated at 37°C. The percent cell death was calculated using uptake into A549 lung cells after 8 h and normalized to lung cells treated with *P. aeruginosa* PAO1 in the absence of EPS273, of which the cell death was set as 100%. All human cell experiments were carried out in accordance with the ethical guidelines of Chinese Academy of Sciences.

### Inhibition Activities of EPS273 on *P. aeruginosa* PAO1 Infection Toward Zebrafish

Wild type zebrafish were maintained at 28°C on a 10 h light/14 h dark cycle and food was freely available. The zebrafish embryos were generated by natural pair-wise mating (3–12 months old) as described before (Westerfield, 1993). Healthy, transparent and regular embryos were selected and aliquotted into 96-well plates with five embryos per well, then incubated at 28°C in 200 μl embryo water. Thereafter, 5 μl of *P. aeruginosa* PAO1 preincubated or non-preincubated with different concentrations of EPS273 for 6 h was added to the embryos at 24 h post-fertilization (hpf). In addition, 5 μl of *P. aeruginosa* PAO1 non-preincubated with EPS273 was also added parallel with different concentrations of EPS273. The zebrafish embryos were further incubated at 28°C, and development of the embryos was observed using an inverted microscope (NIKON TS100, Japan) equipped with a digital camera. All animal experiments were conducted in accordance with the ethical guidelines of Chinese Academy of Sciences.

### Antioxidant Activities of EPS273 by Scavenging Hydroxyl Radical and Superoxide Radical

Hydroxyl radical-scavenging activity of EPS273 was determined according to the method described previously with little modification (Yin et al., 2010). Briefly, 50 μl of PBS (20 mM, pH 7.4), 25 μl of 1,10 - phenanthroline solution (2.5 mM), 25 μl FeSO_4_ solution (2.5 mM), and 25 μl of H_2_O_2_ (20 mM) were added successively in a tube and mixed thoroughly. Then 100 μl of EPS273 aliquot with various concentrations was added in the mixture and incubated at 37°C for 1 h. The absorbance was measured at 536 nm immediately and hydroxyl radical scavenging activity was expressed as Scavenging activity (%) = (As-Ac/Ao-Ac) × 100. Where As is absorbance of the sample in the presence of different concentrations of EPS273, Ac is absorbance of the sample in the absence of EPS273, and Ao is absorbance of the sample without both EPS273 and H_2_O_2_.

Superoxide radical-scavenging activity of EPS273 was performed according to Zhang et al. (2011) with minor modification. An aliquot of 50 μl of Tris-HCl buffer (pH 8.0, 150 mM) was mixed with 25 μl of 1,2,3-phentriol (1.50 mM, dissolved with 10 mM HCl) and 100 μl of EPS273 aliquot with various concentrations. After thorough mixing, the mixture was incubated at room temperature for 30 min. The absorbance of the mixture was measured at 325 nm and superoxide radicals scavenging generated by 1,2,3-phentriol autoxidation was calculated as follows: Scavenging activity (%) = [1- (A_11_-A_10_/A_01_-A_00_)] × 100. Where A_00_ is absorbance of the sample in absence of EPS 273 and 1,2,3-phentriol, A_01_ is absorbance of the sample contained 1,2,3-phentriol but no EPS 273, A_10_ is absorbance of the sample contained EPS 273 but no 1,2,3-phentriol, and A_11_ is absorbance of the sample contained EPS 273 and 1,2,3-phentriol.

### Antibiofouling Activity of EPS273 Tested in the Ocean Environment

The paint containing different concentrations of EPS273 or the same amount EPS273 stocking buffer was coated to the glass slides and dried in the air. Thereafter, the dried glass slides were immersed in the ocean environment of Zhonggang harbor, Qingdao, China. After one or 2 week(s), the glass slides were taken out for imaging and bacteria/diatoms numbers counting. Briefly, the glass slides were rinsed with sterile seawater three times, then the paint sheets were removed completely with knife and put into the tube with sterile seawater. The tubes were vibrated vigorously, and one part of the suspension was diluted in marine broth and plated in marine agar plate for bacteria number counting, the other part was diluted in the sterile seawater and used to count the diatoms numbers under microscope.

### Statistical Analysis

The results were presented as the mean ± SD (standard deviations) and were analyzed by a student’s *t*-test. The values of *P* < 0.05 were used to identify statistically significant differences. All measurements were performed in triplicate.

## Results

### Antibiofilm Activity of *P. stutzeri* 273 Culture Supernatant Against *P. aeruginosa* PAO1

**FIGURE 1:**
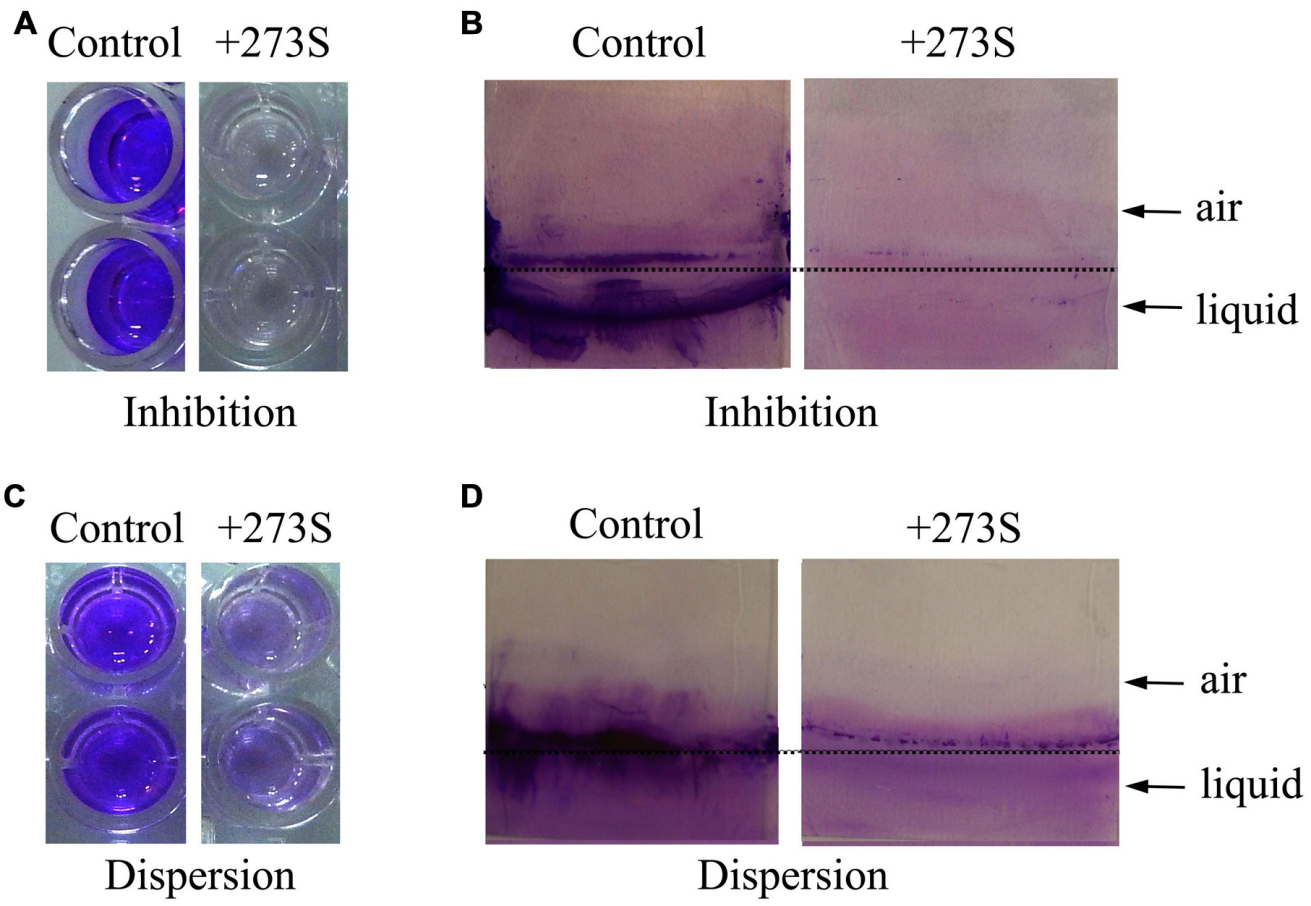
**Effects of *Pseudomonas stutzeri* 273 supernatant on biofilm formation and dispersion of *P. aeruginosa* PAO1. (A)** Biofilm formation of *P. aeruginosa* PAO1 in 96-well polystyrene plate wells in the presence or absence of 10% supernatant of *P. stutzeri* 273 (273S) and duplicate wells are shown. **(B)** Biofilm formation of *P. aeruginosa* PAO1 on glass slides in the presence or absence of 10% 273S. As for the controls, the same amount of LB broth was used. **(C)** Preformed biofilm dispersion of *P. aeruginosa* PAO1 in 96-well polystyrene plate wells in the presence or absence of 10% 273S and duplicate wells are shown. **(D)** Preformed biofilm dispersion of *P. aeruginosa* PAO1 on glass slides in the presence or absence of 10% 273S. As for the controls, the same amount of LB broth was used.

In order to obtain potential biofilm formation inhibitors, the culture supernatants of more than 400 marine bacterial strains were screened and evaluated by their abilities to inhibit biofilm formation by *P. aeruginosa* PAO1 using 96-well polystyrene plate. Among them, the strain 273 exhibited the strongest ability to inhibit biofilm formation. As shown in **Figure 1A**, the supernatant of strain 273 almost abolished the biofilm formation completely at the dilution of 1/10, which was further confirmed by the analysis on glass slides as shown in **Figure 1B**.

While the culture supernatant of marine bacterium strain 273 could greatly inhibit biofilm formation by *P. aeruginosa* PAO1, we sought to explore whether the culture supernatant could disperse the preformed biofilm in the 96-well polystyrene plate. This assay was performed by shifting the preformed biofilms to medium supplemented with culture supernatant of bacterium strain 273 at the dilution of 1/10, and the fate of biofilms was measured after co-incubation overnight. As shown in **Figure 1C**, the culture supernatant of bacterium strain 273 also had strong ability to disperse the established biofilms when diluted to 1/10, which was also confirmed by analysis on glass slides (**Figure 1D**).

Based on above results, we concluded that the culture supernatant of marine bacterium strain 273 had strong antibiofilm activity, which was not restricted to inhibition of biofilm formation, but also effective to disperse mature biofilm by *P. aeruginosa* PAO1. According to the high homology (99% identity) with marine bacterium *P. stutzeri* by the 16S rRNA gene sequencing (Accession no. KU529971), the bacterium strain 273 was designated as *P. stutzeri* 273.

### Purification and Identification of Antibiofilm Component EPS273 from *P. stutzeri* 273

**FIGURE 2:**
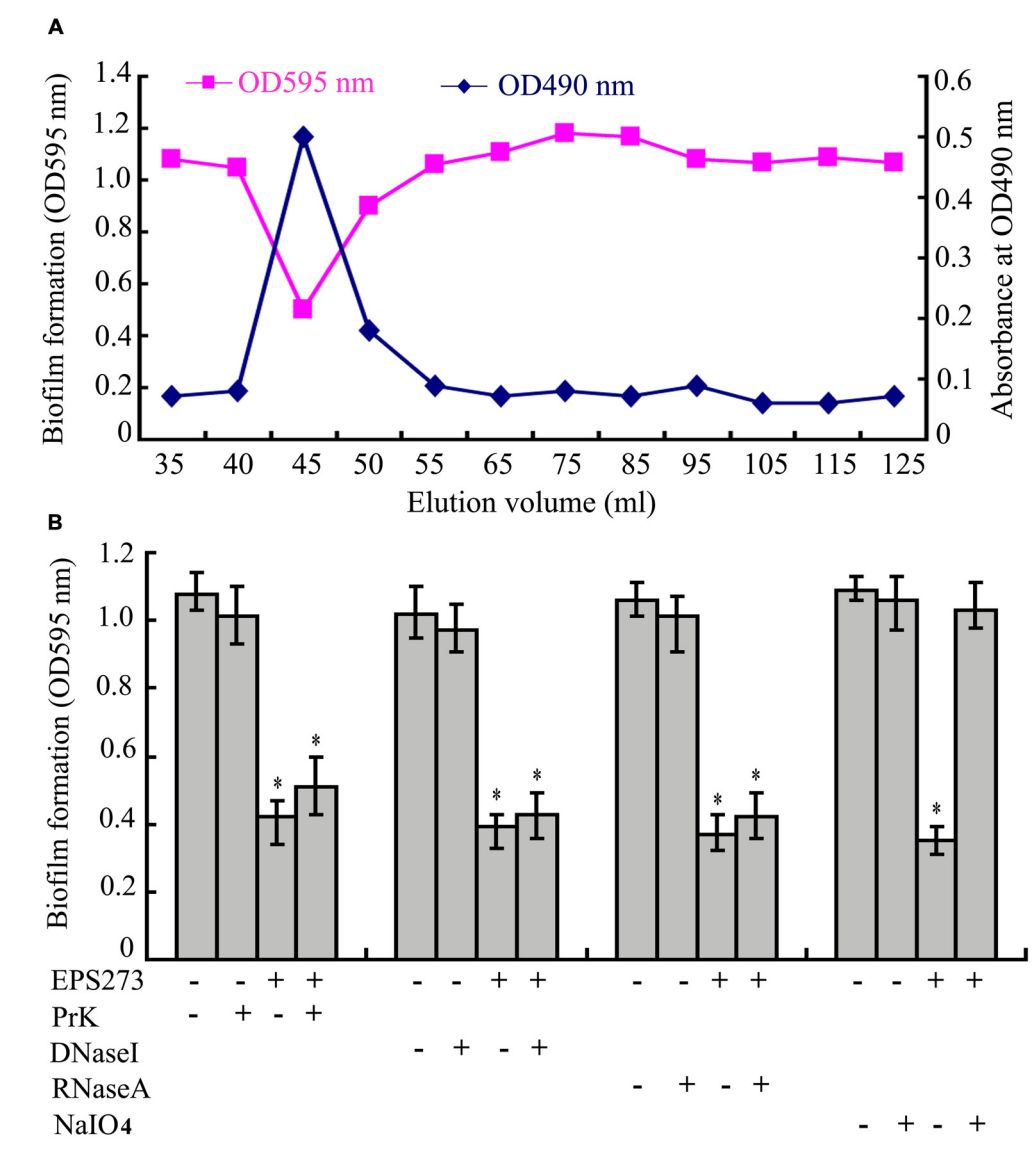
**Purification and identification of active component of EPS273. (A)** The profiles of the fractions in the gel filtration, which were collected and monitored for the biofilm formation determined at OD595 nm after crystal violet staining and polysaccharide content determined at OD490 nm after the phenol-sulfuric acid assay. **(B)** Effects of Proteinase K, DNase I, RNase A and NaIO_4_ on the activities of EPS273 inhibiting biofilm formation of *P. aeruginosa* PAO1. EPS273 (0.1 μg/mL) was, respectively, treated with proteinase K (PrK) (1 mg/mL), DNaseI (100 μg/mL), RNaseA (100 μg/mL) for 1 h or NaIO_4_ (10 mM) for 12 h at 37°C, then taken to measure the antibiofilm activity. Error bars represent standard deviations of three independent experiments. Error bars indicate the standard deviations of three measurements. ^∗^*P* < 0.05 versus the control.

To elucidate the active component inhibiting biofilm formation by *P. aeruginosa* PAO1, ammonium sulfate precipitation, dialysis, anion exchange and gel filtration were applied to purify the active component from the supernatant of *P.* 273. Surprisingly, the active component was eluted immediately after the void volume on Superdex^TM^ 200 column (**Figure 2A**), which indicated that the antibiofilm component of *P. stutzeri* 273 supernatant had a high molecular weight. Due to its high molecular weight, we proposed that the active fraction might be polysaccharide-related macromolecule. Therefore, the polysaccharide content in the elution fractions was checked by the phenol-sulfuric acid method. As expected, the antibiofilm activity was positively related to polysaccharide concentration (**Figure 2A**), which suggested that the effective component of *P. stutzeri* 273 might be a high-molecular-mass polysaccharide.

To further confirm this speculation, proteinase K, DNase I, RNase A and NaIO4, were used to digest the purified active component, respectively. As shown in **Figure 2B**, treatment of the active component with proteinase K, DNase I and RNase A had no effect on its antibiofilm activity. In contrast, treatment of the active component with NaIO_4_ significantly reduced its antibiofilm activity. It is well known that NaIO_4_ is able to hydrolyze polysaccharides by oxidizing the carbons bearing vicinal hydroxyl groups and cleaving the C-C bonds (Mack et al., 1996; Jiang et al., 2011). Taken together, the characteristics of antibiofilm component indicated that it could be a high-molecular-weight polysaccharide. Therefore, the active component from bacterium strain *P. stutzeri* 273 was named as exopolysaccharide 273 (EPS273).

### Functional Group and Monosaccharide Composition Analyses of EPS273

**FIGURE 3:**
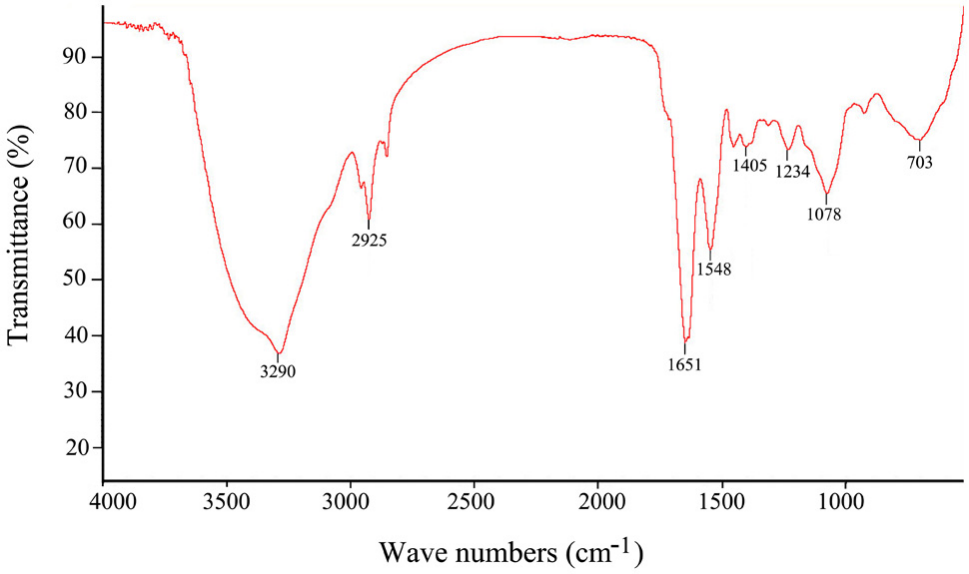
**Fourier transform infrared spectrum of the purified EPS273 from *P. stutzeri* 273 in the range 500–4000 cm^-1^**.

The major functional groups of EPS273 were determined by FT-IR spectroscopy, indicating a typical pattern of absorption for a polysaccharide FT-IR of EPS273 revealed typical characteristics of polysaccharide (**Figure 3**). The stretching vibration at 3290 cm^-1^ was ascribed to the hydroxyl group and the weak peak at 2925 cm^-1^ was attributed to an asymmetrical C-H stretching vibration of the aliphatic CH_2_ group, which indicates the presence of organic substances such as proteins, sugars etc. (Wang et al., 2015). The absorption at 1651 cm^-1^ resembled the ring stretching of mannose (Wang et al., 2015). The relatively weak absorption peak at 1548 cm^-1^ might be attributable to the N-H bending of amides II of proteins (Lin et al., 2005). The absorbance peak at 1405 cm^-1^ was possibly due to the symmetric stretching of the –COO^-^ group (Jiang et al., 2011). The absorption peaks within 1200–1000 region were assigned to the vibrations of the C-O and C-O-C glycosidic bands (Wang et al., 2015). The absorption peak at 1078 indicated the presence of a polysaccharide (Wang et al., 2015).

High-performance liquid chromatography traces of the polysaccharide hydrolyzate showed monosaccharide components of EPS273. The predominant monosaccharides of EPS273 were glucosamine (35.4%), rhamnose (28.6%), and glucose (27.2%). Besides the three abundant monosaccharides, mannose was also detected at relatively low level (8.7%). Additional, HPGPC was applied to elucidate the molecular weight of the polysaccharide and the average molecular weight is about 190 kDa.

### Fluorescence Microscopic Observation of Biofilm Inhibition and Dispersion by EPS273

**FIGURE 4:**
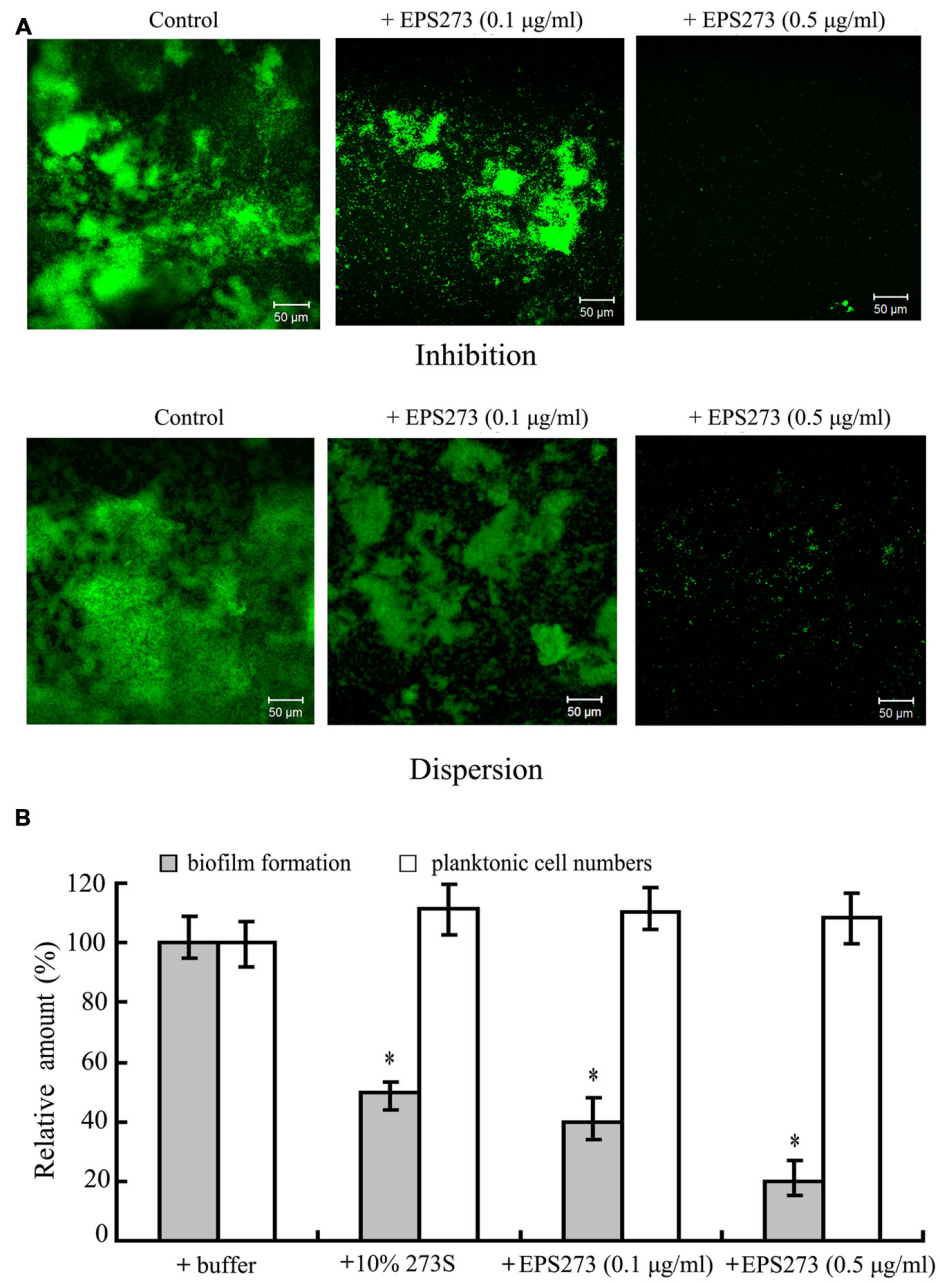
**Anbibiofilm and antibacterial activity analyses of purified EPS273. (A)** Florescence microscopic observation of inhibition on biofilm formation and dispersion on preformed biofilm by EPS273. The images are representative of three separate experiments. **(B)** Antibacterial activity analyses of EPS273. Error bars indicate the standard deviations of three measurements. ^∗^*P* < 0.05 versus the control.

Since the culture supernatant of *P. stutzeri* 273 could greatly inhibit biofilm formation and disperse the preformed biofilm of *P. aeruginosa* PAO1, we also checked whether the purified EPS273 had the similar function. As shown in **Figure 4A**, the biofilm formation or the preformed biofilm on glass slides were greatly reduced at the concentration of 0.1 μg/mL purified EPS273, and almost abolished at the concentration of 0.5 μg/mL, which was consistent with our above results obtained from the culture supernatant of *P. stutzeri* 273.

### Antibiofilm and Antibacterial Assays of EPS273 Against *P. aeruginosa* PAO1

Consistent findings of antibiofilm activity were also observed in 96-well polystyrene with the purified EPS273. As shown in **Figure 4B**, the biofilm formation was gradually reduced as the concentration of the purified EPS273 was increased from 0.1 to 0.5 μg/mL. In order to check whether the antibiofilm activity was related with the antimicrobial activity, the planktonic cells of the wells supplemented with or without purified EPS273 were also enumerated. As shown **Figure 4B**, the cells in the planktonic culture were hardly changed even the concentration of purified EPS273 was up to 0.5 μg/mL. Based on above results, we concluded that the antibiofilm activity of purified EPS273 did not result from reducing bacterial growth.

### Inhibition Activities of EPS273 on Intercellular Adhesion, Production of Virulence Factors, H_2_O_2_ and eDNA by *P. aeruginosa* PAO1

**FIGURE 5:**
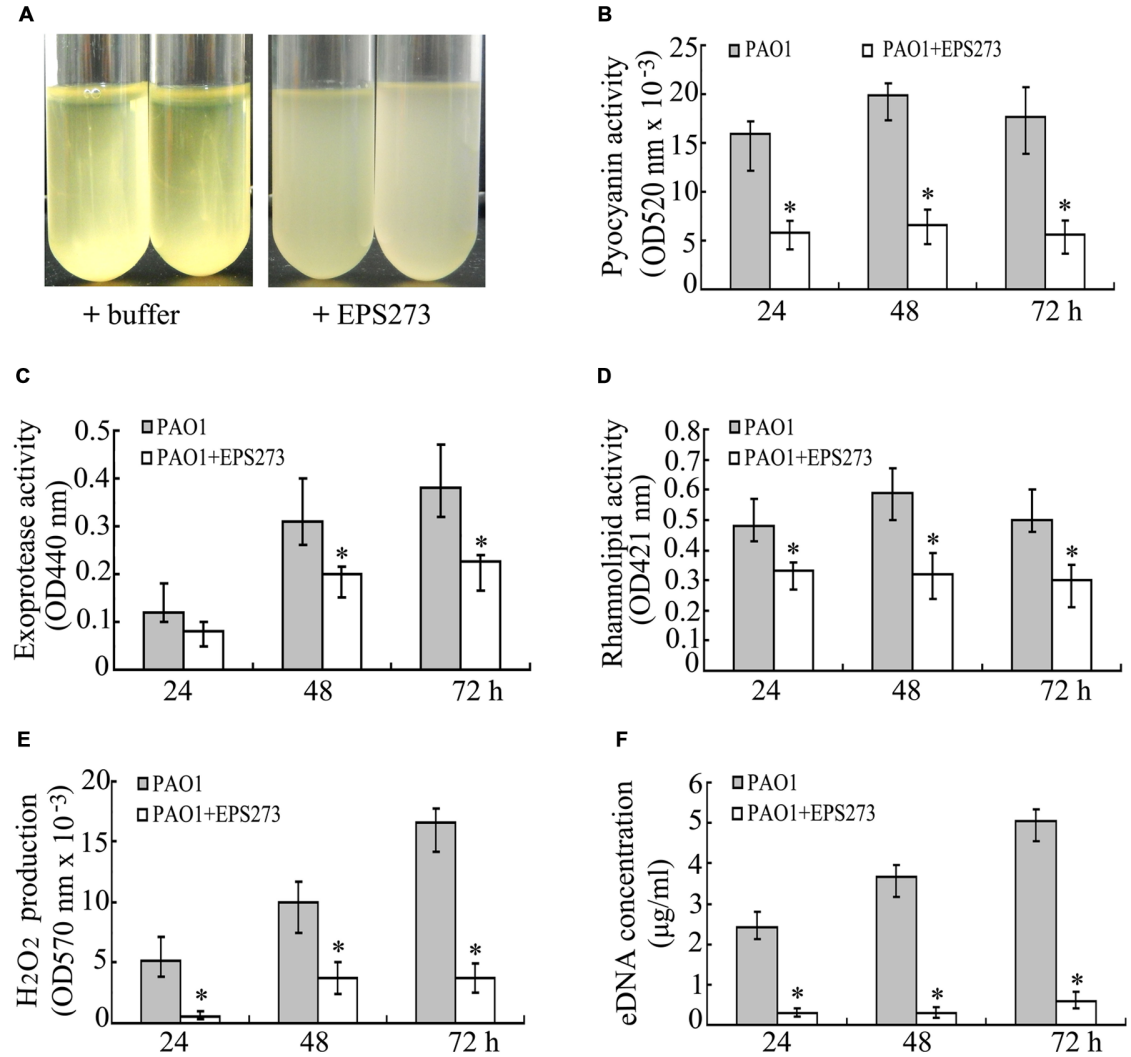
**Effects of EPS273 on intercellular adhesion **(A)**, the production of pyocyanin **(B)**, exoprotease **(C)**, rhamnolipid **(D)**, hydrogen peroxide (H_2_O_2_) **(E)** and extracellular DNA (eDNA) **(F)** of *P. aeruginosa* PAO1.** The concentration of EPS273 used in the above assays is 0.1 μg/mL. Error bars indicate the standard deviations of three measurements. ^∗^*P* < 0.05 versus the control.

It is well established that intercellular adhesions are critical for bacterial biofilm formation. To investigate the effect of EPS273 on the cell surface interaction, *P. aeruginosa* PAO1 cells were cultured in LB broth with or without EPS273 at 37°C for 24 h. As shown in **Figure 5A**, the cells cultured in broth without EPS273 aggregated and settled to the bottom of the tube, resulting in a visible clearing of the broth. In contrast, cells cultured in broth containing EPS273 exhibited less settling and more cloudy. These data suggest that EPS273 can inhibit the intercellular adhesion by *P. aeruginosa* PAO1.

Pyocyanin is one of the most important factors for infection and biofilm formation by *P. aeruginosa* PAO1 (Das and Manefield, 2012). Next we sought to investigate the production of pyocyanin by *P. aeruginosa* PAO1 with or without EPS273 treatment. As shown in **Figure 5B**, the production of pyocyanin in cells treated with EPS273 was greatly decreased compared with cells without EPS273 treatment. Since EPS273 inhibited pyocyanin production in *P. aeruginose* PAO1, we also investigated whether EPS273 could inhibit other virulence factors, such as exoprotease and rhamnolipid. As shown in **Figures 5C,D**, production of exoprotease and rhamnolipid was also reduced by EPS273, even when it was coincubated with *P. aeruginose* PAO1 up to 72 h.

It is reported that production of pyocyanin promotes biofilm formation in *P. aeruginosa* via eDNA release through H_2_O_2_ mediated cell lysis (Das and Manefield, 2012). Since EPS273 can inhibit pyocyanin production greatly, it is worth to investigate simultaneously whether EPS273 can affect H_2_O_2_ generation and eDNA release in *P. aeruginosa* PAO1. As expected, EPS273 also dramatically inhibited H_2_O_2_ generation (**Figure 5E**) and eDNA release (**Figure 5F**) of *P. aeruginosa* PAO1 up to 72 h. Based on above results, we proposed that one of the main reasons of antibiofilm activity of EPS273 might be due to its inhibition activity on H_2_O_2_ and eDNA release through decreasing pyocyanin production.

### Inhibition Activities of EPS273 on *P. aeruginosa* PAO1 Infection to Human Lung Carcinosis A549 Cells and Zebrafish Embryos

**FIGURE 6:**
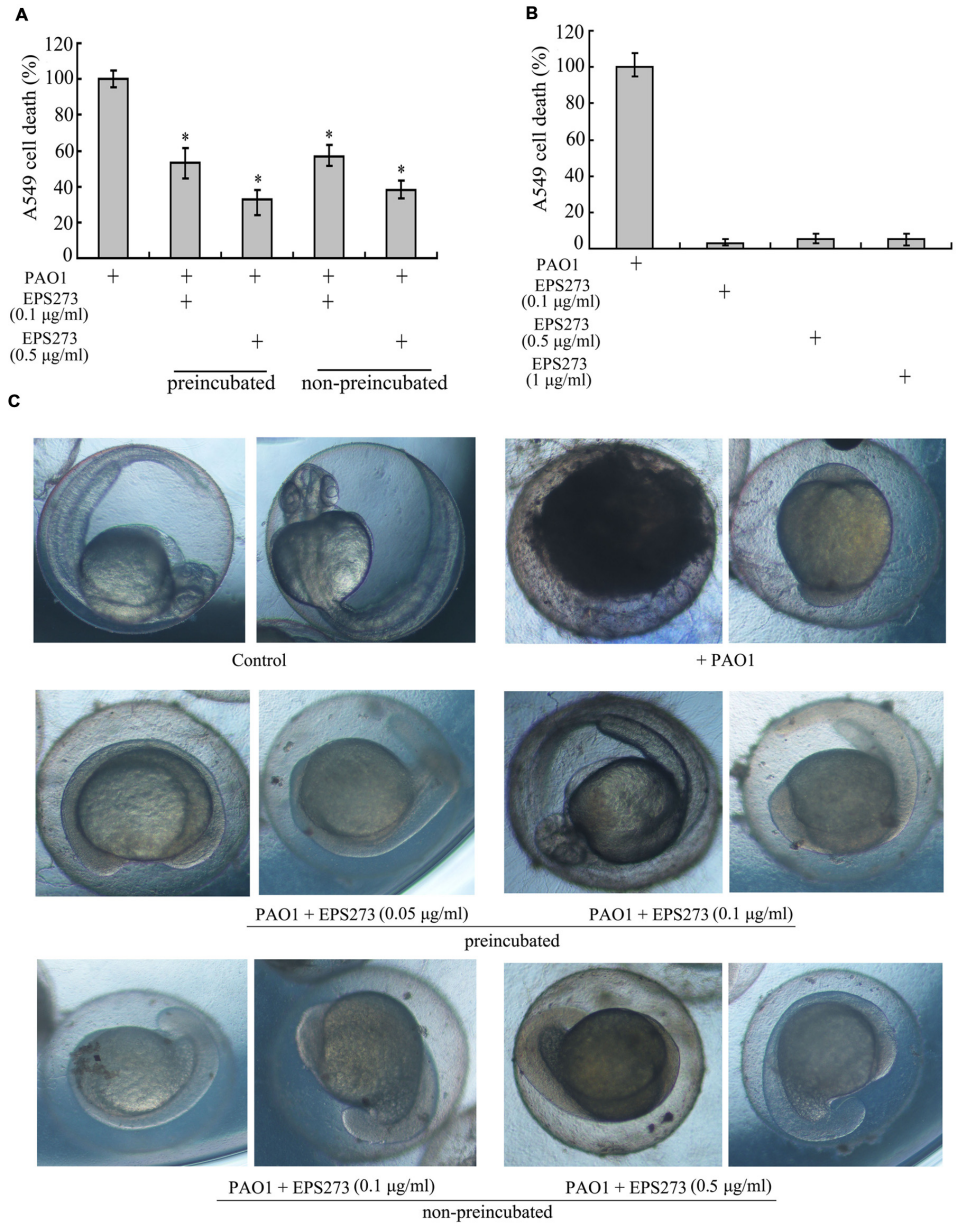
**Inhibition activities of EPS273 on *P. aeruginosa* PAO1 infection to human A549 lung cells and zebrafish embryos. (A)** EPS273 inhibits *P. aeruginosa* PAO1 infection to human A549 lung cells. The percent cell death was calculated using propidium iodide uptake into A549 lung cells after 8 h and normalized to cells in the absence of EPS273, of which the cell death was set as 100%. Error bars indicate the standard deviations of three measurements. ^∗^*P* < 0.05 versus the control. **(B)** The cytotoxic analysis of EPS273 to A549 human lung cells. A549 human lung cells were treated with 0.1, 0.5, and 1 μg/mL of EPS273 in the absence of *P. aeruginosa* PAO1. The percent cell death was calculated using uptake into A549 lung cells after 8 h and normalized to lung cells treated with *P. aeruginosa* PAO1 in the absence of EPS273, of which the cell death was set as 100%. Error bars indicate the standard deviations of three measurements. ^∗^*P* < 0.05 versus the control. **(C)** EPS273 inhibits *P. aeruginosa* PAO1 infection to zebrafish embryos. Zebrafish embryos were infected with *P. aeruginosa* PAO1 in the presence or absence of EPS273 and imaged after 24 h infection. All the pictures were taken with inverted microscope (Nikon TS100, Japan) at 40X.

It is well known that *P. aeruginosa* is regarded as a serious pathogen to chronic lung infection in cystic fibrosis patient, and pyocyanin is an important virulence factor during this infection (Lau et al., 2004). Since EPS273 inhibited pyocyanin production in *P. aeruginosa* PAO1, we next asked whether EPS273 could depress *P. aeruginosa* infection to human lung cells. To answer this question, we tested the cell death of human lung cell line A549 by *P. aeruginosa* PAO1 infection in the absence or presence of EPS273. As shown in **Figure 6A**, the cell death was reduced gradually (from 80 to 60%) if we preincubated *P. aeruginosa* PAO1 with increased concentration of EPS273 (from 0.05 to 0.1 μg/mL) before its infection. Moreover, the cell death by *P. aeruginosa* PAO1 infection was also reduced greatly if we treated *P. aeruginosa* PAO1 with EPS273 at the same time of its infection. The assays clearly showed that *P. aeruginosa* PAO1 were capable of killing A549 cells, while EPS273 decreased the killing rate regardless of preincubated or simultaneously treated *P. aeruginosa* with EPS273 during the infection. Meanwhile, EPS273 is not toxic to the human cells even in high concentration (**Figure 6B**). Therefore, EPS273 could attenuate *P. aeruginosa* PAO1 infection to human lung cell A549.

Recently, the zebrafish (Danio rerio) has become an important vertebrate animal model to study the pathogen infection, especially in its embryotic stage (Goldsmith and Jobin, 2012). In this study, we also investigated whether EPS273 could attenuate the infection of *P. aeruginosa* PAO1 to zebrafish embryos. As shown in **Figure 6C**, the zebrafish embryos developed very well in the absence of *P. aeruginosa* PAO1, while the embryos almost terminated the development when infected by *P. aeruginosa* PAO1. However, if we preincubated *P. aeruginosa* PAO1 with EPS273 before infection or treated *P. aeruginosa* PAO1 with EPS273 at the same time of its infection, the zebrafish embryos developed much better compared with those infected by *P. aeruginosa* PAO1 without EPS273 treatment. Collectively, we concluded that EPS273 could reduce *P. aeruginosa* PAO1 infection to zebrafish embryos.

### Antioxidant Activities of EPS273 on Hydroxyl Radical and Superoxide Radical

**FIGURE 7:**
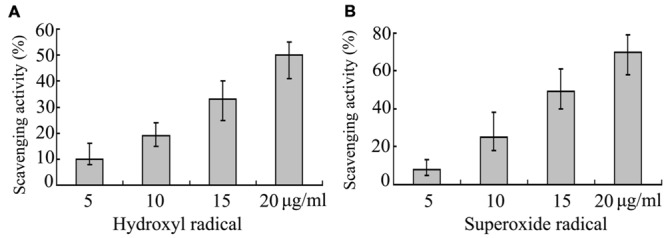
**Scavenging activities of EPS273 on hydroxyl radical **(A)** and superoxide radical (B).** Error bars represent standard deviations of three independent experiments. Error bars indicate the standard deviations of three measurements.

EPS273 could effectively inhibit the biofilm formation and infection of *P. aeruginosa* PAO1 to mamallain cells and zebrafish. We next sought to check the antioxidant activity of EPS273. Antioxidant activities have been performed with different reaction mechanisms including free radical scavenging, reductive capacity, binding of transition metal ion catalysts and inhibition of chain initiation (Frankel and Meyer, 2000). Among reactive oxygen species, hydroxyl radical is the most reactive in chemistry and it can react with all biomacromolecules in living cells resulting in severe damage to the adjacent macromolecules (Zhang et al., 2013). Therefore, the ability of EPS273 to scavenge hydroxyl radicals was investigated. As shown in **Figure 7A**, EPS273 exhibited concentration-dependent scavenging activities against hydroxyl radicals and could scavenge hydroxyl radicals up to 50% at concentration of 60 μg/mL.

Superoxide anions are active free radical precursors that have capacity to act in response to biological macromolecules and damage tissues through oxidative damage (Kanmani et al., 2011). In addition, the vital role of superoxide radicals in the formation of hydrogen peroxide, hydroxyl radical and single oxygen that can induce oxidative damage in lipids, proteins and DNA (Wickens, 2001). Therefore, the ability of EPS273 to scavenge superoxide radicals was investigated. As shown in **Figure 7B**, EPS273 also exhibited concentration-dependent scavenging activities against superoxide radicals which were similar to that of hydroxyl radicals. EPS273 could scavenge hydroxyl radicals up to 70% at concentration of 60 μg/mL (**Figure 7B**).

Collectively, together with the potential antioxidant activity, non-toxic to human cells and biofilm inhibiting properties of EPS273, we proposed that EPS273 might have the potential applications in food and heath care related fields.

### Antibiofouling Activity of EPS273 in the Marine Environment

**FIGURE 8:**
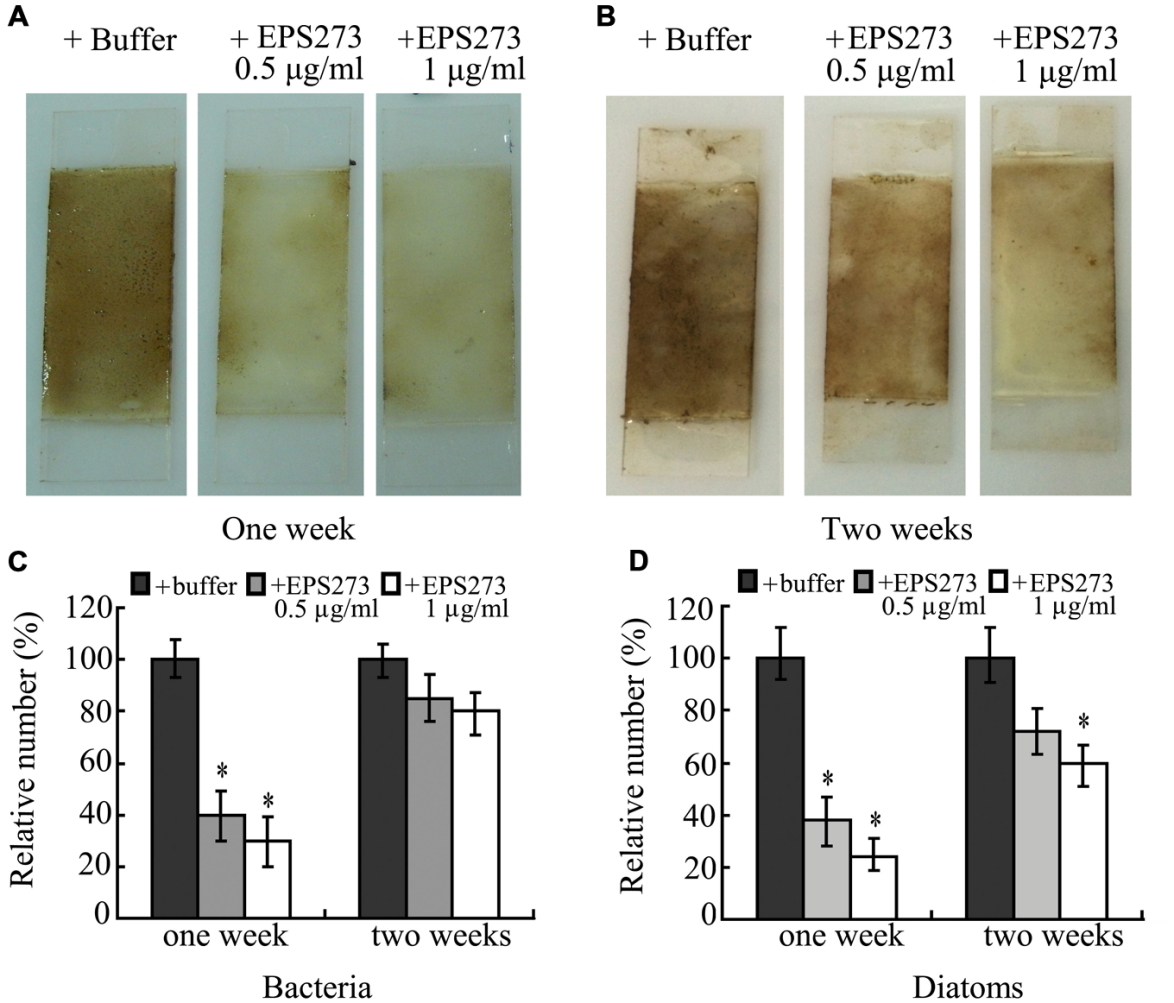
**Antibiofouling activity of EPS273 in marine environment. (A)** and **(B)** are images of biofouling formation on glass slides precoated by paints with or without EPS273 after 1 or 2 weeks in the ocean environment, respectively. **(C)** and **(D)** are the relative amounts of bacteria and diatoms from corresponding glass slides, respectively. The amount of bacteria or diatoms from glass slides without EPS273 was set at 100%. Error bars indicate the standard deviations of three measurements. ^∗^*P* < 0.05 versus the control.

For the aquaculturist, the effects of biofouling are largely detrimental (Braithwaite et al., 2007). The most common way to prevent or delay biofouling in marine aquaculture is to coat the submerged structures and net-cages with antifouling paints including copper and zinc (Cotou et al., 2012), which have lethal or sub-lethal effects on farmed fish and could affect the immediate immune defense mechanism of the exposed fish (Cotou et al., 2012). Therefore, an environment-friendly antifouling paint additive is urgently needed. Based on our results, marine-derived exopolysaccharide EPS273 showed excellent antibiofilm characteristics in the laboratory. Next, we asked whether this exopolysaccharide was still functional well in the opening marine environment. To answer this question, the antifouling abilities of paints with or without EPS273 were tested in the ocean. The results showed that EPS273 could effectively inhibit the biofouling in the first week (**Figure 8A**) and the antibiofouling activity decreased gradually in the second week (**Figure 8B**). Accordingly, the amounts of bacteria and diatoms decreased dramatically in the first week with the adding of EPS273 (**Figures 8C,D**), while the amounts of bacteria and diatoms increased in the second week compared to those of the first week, which indicated that the antibiofouling activity of EPS273 decreased gradually as time went on in the seawater. Therefore, how to keep antibiofouling activity of EPS273 for longer time in the marine is our endower to solve in the next step.

## Discussion

Biofilm formation is a lifestyle commonly adopted by a variety of microorganisms whether in the environment or in clinical settings (Gnanadhas et al., 2015). Within the biofilm, pathogenic microorganisms are refractory to antibiotics, requiring concentrations up to 1000-fold more resistant to antibiotic treatment than the same organism growing planktonically (Simoes et al., 2009). *P. aeruginosa*, a notorious opportunistic and nosocomial pathogenic bacterium, can form biofilms on a variety of natural and man-made environments such as natural water bodies, soil, skin, and medical/food devices (Costerton, 2001). The ability of biofilm-embedded bacteria to resist clearance by antimicrobial agents points to the importance of a continuous search for novel molecules with new mechanisms of action that are effective against bacterial biofilm, which will be helpful to solve the alarming ability of *P. aeruginosa* to resist antibiotics (Mesaros et al., 2007).

Normally, EPS is a common component of biofilm and its production is an important feature of the mature biofilm (Fux et al., 2003). In many bacteria, increased biofilm formation often correlates with increased EPS production (Jiang et al., 2011). However, few bacterial EPSs were recently found to negatively regulate biofilm formation. The first antibiofilm polysaccharide was discovered while studying interaction between uropathogenic and commensal strains of *Escherichia coli* in mixed *in vitro* biofilm (Valle et al., 2006). Later studies suggest that some bacterial exopolysaccharides can perform functions that inhibit or destabilize the biofilm (Jiang et al., 2011; Rendueles et al., 2013). In this study, we discovered a marine bacterium exopolysaccharide EPS273 that not only effectively reduced *P. aeruginosa* PAO1 biofilm formation but also dispersed the preformed biofilm. Furthermore, it inhibited the production of virulence factor pyocyanin, thus leading to greatly attenuated infection of *P. aeruginosa* PAO1 to human lung cells and zebrafish embryos.

EPS273 doesn’t exhibit bactericidal activity. Its antibiofilm activity, therefore, is likely to be mediated by mechanisms other than growth inhibition. As known that biofilm persistence is attributed to a matrix of extracellular polymeric substances made up of polysaccharides, proteins, lipids, and extracellular DNA (eDNA; Flemming and Wingender, 2010). A decade ago, Whitchurch et al. (2002) discovered that eDNA is major component of biofilms formed by *P. aeruginosa* and is essential for biofilm formation and stability. Recently, it has been reported that eDNA release in *P. aeruginosa* is promoted by pyocyanin through the cell lysis mediated by H_2_O_2_ generation (Das and Manefield, 2012). Furthermore, it is also reported that pyocyanin binds to DNA and increases solution viscosity therefore unveils a novel molecular interaction that can be used to target and control *P. aeruginosa*-mediated biofilm formation and its related lung infection (Das et al., 2015). Our findings showed that EPS273 inhibited not only the production of the virulence factor pyocyanin but also the generation of H_2_O_2_ and eDNA in *P. aeruginosa* PAO1. Along with its antibiofilm characteristic, we speculated that EPS273 might inhibit the biofilm formation by *P. aeruginosa* PAO1 via decreasing the production of pyocyanin, then consequently inhibiting the generation of H_2_O_2_ and release of eDNA, which are essential for biofilm formation and stability. To our best knowledge, this is the first cut-in to reveal the antibiofilm mechanism of marine bacterium exopolysaccharide.

One of the notable characteristic of EPS273 is that it can effectively disperse the preformed biofilm (**Figures 1** and **4**). Several different mechanisms have been implicated in the biofilm dispersion, including cell death, matrix-degrading enzymes, and induction of cellular motility (Karatan and Watnick, 2009; Kaplan, 2010). Several antibiofilm polysaccharides have also been shown to enhance or trigger biofilm dispersal (Bendaoud et al., 2011; Jiang et al., 2011). In spite of recent research on the mechanisms of action of antibiofilm polysaccharides and other biosurfactants, the precise mechanisms by which they break-down preformed biofilms are yet to be elucidated (Rendueles et al., 2013). Therefore, it will be very interesting to reveal the detailed antibiofilm mechanisms of EPS273 in the future.

In recent years, polysaccharides from bacteria, yeast, fungi, and medicinal plants have been reported to possess antioxidant activities and could be used as natural antioxidant for the development of effective and non-toxic medicines with stronger antioxidant activities *in vitro* and *in vivo* (Zhang et al., 2013; Li et al., 2014). Notably, it is reported that exopolysaccharides isolated from *Acinetobacter* sp., *Streptococcus* sp., and *Pseudomonas* sp. have been used for food and health care products (Kumar et al., 2007; Delbarre-Ladrat et al., 2014). Furthermore, in the last decade, several products from *P. stutzeri, P. fluorescens*, and *P. amyloderamosa* have successfully entered the Generally Recognized as Safe (GRAS) Notice Inventory^2^. Our findings showed that EPS273 exhibited strong antioxidant potential by quenching hydroxyl and superoxide anion radicals, and also protected human lung epithelial cells and zebrafish embryos from killing by *P. aeruginosa* PAO1. As we mentioned before, *P. aeruginosa* is regarded as a notorious nosocomial pathogen and a major food spoilage microorganism in food processing industry, along with the strong antibiofilm activity of EPS273 against *P. aeruginosa*, we believe that EPS273 might have the potential to be used in the food and healthcare industry.

The frequent occurrence of biofouling in marine aquaculture is a significant challenge resulting in increased operational expenses and deleterious impacts on the species being cultured (Fitridge et al., 2012). The control of biofouling in aquaculture is achieved through using copper/zinc containing antifouling paint. However, the continued increasingly stringent legislation for biocides in food production necessitates the development of environmental and societal antifouling agents effectively preventing the settlement and growth of resilient multi-species consortia of biofouling organisms in the opening ocean environment (Fitridge et al., 2012). Unfortunately, marine condition is much more complicated than that in the laboratory, which makes most of the environmental friendly antibiofouling compounds lose their function when tested in the ocean. Notably, EPS273 shows great antibiofouling effects to both bacteria and diatoms up to 2 weeks, which indicates that EPS273 maybe suitable to develop environmental friendly antibiofouling agents in aquaculture.

It is reported that the biofilm formation, the virulence factors production and the antioxidants are closely related (Das and Manefield, 2012; Das et al., 2015). In our study, just as Das et al. (2015) reported, the exopolysaccharide EPS273 not only could inhibit biofilm formation and disperse preformed biofilm, but also has inhibition activity on the production of virulence factors and also has antioxidant activity by scavenging of free radicals. Therefore, these properties of EPS273 make it potential for the treatment and prevention of biofilm-related infection, food-processing contamination and the biofouling in marine aquaculture.

## Author Contributions

SW and CS conceived and designed the experiments. SW performed most of the experiments. GL helped to do and analyze the experiments about mamallian cells and zebrafish. WJ helped to perform the physical and chemical analyses of EPS273. PX helped to purify the EPS273. SW, GL, and CS analyzed the data. SW and CS prepared the figures and wrote the paper. All authors reviewed the manuscript.

## Conflict of Interest Statement

The authors declare that the research was conducted in the absence of any commercial or financial relationships that could be construed as a potential conflict of interest.

## Footnotes

1 http://blast.ncbi.nlm.nih.gov/Blast.cgi

2 http://www.accessdata.fda.gov/scripts/fdcc/?set=GRASNotices
